# Supplementary material for: Global hypo-methylation in a proportion of glioblastoma enriched for an astrocytic signature is associated with increased invasion and altered immune landscape
Source: eLife. 2022 Nov 22;11:e77335. doi: 10.7554/eLife.77335 (PMC9681209; doi:10.7554/eLife.77335)
Supplement: Figure 2—source data 1. [file elife-77335-fig2-data1.zip › Figure_2_source_data_1/Figure_2A/homerResults/motif63.similar.html]

motif63

## Information for motif63

T
C
A
G
A
C
T
G
A
C
G
T
C
G
T
A
A
G
T
C
A
G
T
C
C
G
T
A
A
G
T
C
  
Reverse Opposite:  

A
C
T
G
A
C
G
T
A
C
T
G
A
C
T
G
A
C
G
T
C
G
T
A
A
G
T
C
A
G
T
C
  

|  |  |
| --- | --- |
| p-value: | 1e-10 |
| log p-value: | -2.468e+01 |
| Information Content per bp: | 1.929 |
| Number of Target Sequences with motif | 28.0 |
| Percentage of Target Sequences with motif | 1.21% |
| Number of Background Sequences with motif | 9.8 |
| Percentage of Background Sequences with motif | 0.27% |
| Average Position of motif in Targets | 74.2 +/- 48.1bp |
| Average Position of motif in Background | 107.7 +/- 66.4bp |
| Strand Bias (log2 ratio + to - strand density) | 0.7 |
| Multiplicity (# of sites on avg that occur together) | 1.00 |
| Motif File: | file (matrix) reverse opposite |

### Similar de novo motifs found

|  |  |  |  |  |  |  |  |
| --- | --- | --- | --- | --- | --- | --- | --- |
| Rank | Match Score | Redundant Motif | P-value | log P-value | % of Targets | % of Background | Motif file |
| 1 | 0.641 | A G T C C G T A A C G T A C T G A C T G A C G T G T A C A G T C | 1e-9 | -22.602249 | 1.51% | 0.41% | motif file (matrix) |
| 2 | 0.890 | C T A G A C G T C G T A A G T C A T G C C G T A | 1e-8 | -20.014842 | 17.44% | 13.13% | motif file (matrix) |
